# Single Fascia Iliaca Compartment Block is Safe and Effective for Emergency Pain Relief in Hip-fracture Patients

**DOI:** 10.5811/westjem.2015.10.28270

**Published:** 2015-12-14

**Authors:** Leonieke Groot, Lea M. Dijksman, Maarten P. Simons, Mariska M.S. Zwartsenburg, Jasper R. Rebel

**Affiliations:** Onze Lieve Vrouwe Gasthuis, Department of Emergency Medicine, Amsterdam, Netherlands

## Abstract

**Introduction:**

Currently, it is common practice in the emergency department (ED) for pain relief in hip-fracture patients to administer pain medication, commonly systemic opioids. However, with these pain medications come a high risk of side effects, especially in elderly patients. This study investigated the safety profile and success rate of fascia iliaca compartment block (FICB) in a busy ED. This ED was staffed with emergency physicians (EPs) and residents of varying levels of experience. This study followed patients’ pain levels at various hourly intervals up to eight hours post procedure.

**Methods:**

Between September 2012 and July 2013, we performed a prospective pilot study on hip-fracture patients who were admitted to the ED of a teaching hospital in the Netherlands. These patients were followed and evaluated post FICB for pain relief. Secondary outcome was the use of opioids as rescue medication.

**Results:**

Of the 43 patients in this study, patients overall experienced less pain after the FICB (p=0.04). This reduction in pain was studied in conjunction with the use and non-use of opioids. A clinically meaningful decrease in pain was achieved after 30 minutes in 62% of patients (54% with the use of opioids, 8% without opioids); after 240 minutes in 82% of patients (18% with opioids, 64% without opioids); after 480 minutes in 88% of patients (16% with opioids, 72% without opioids). No adverse events were reported.

**Conclusion:**

In a busy Dutch ED with rotating residents of varying levels of experience, FICB seems to be an efficient, safe and practical method for pain reduction in patients with a hip fracture. Even without the use of opioids, pain reduction was achieved in 64% of patients after four hours and in 72% of patients after eight hours.

## INTRODUCTION

In current emergency department (ED) practice, pain management in hip-fracture patients is hampered by a high risk of side effects. These side effects are particularly noticeable in elderly patients. Twenty-four hours after a hip fracture, 50% of patients aged 50 and up reported “severe to very severe” pain.^1^ To control this pain, nonsteroidal anti-inflammatory drugs (NSAIDs), with or without acetaminophen, were usually not effective. NSAIDs can have a negative impact on renal function, on the mucosa of the gastrointestinal tract and on platelet aggregation. For these and other reasons, pain management in the ED of the Onze Lieve Vrouwe Gasthuis (OLVG), a Dutch teaching hospital in Amsterdam, is based on systemic opioids such as fentanyl and morphine. However, opioids also have a large potential for side effects. Intravascular administration of opioids can lead to nausea and vomiting. Other common side effects of opioids include sedation, respiratory depression and possible delirium. Moreover, many patients will receive suboptimal pain management, which is another risk factor for delirium.^2^,^3^

One of the goals of the ED in the OLVG is safe, rapid and effective pain management to insure avoidable complications. To achieve this, fascia iliaca compartment block (FICB) can be a good option. FICB using the “two pop technique” is found to be safe and relatively easy to perform.^4^–^12^ Anesthesiologists have performed this block for perioperative pain relief with good results in a controlled environment.^5^–^7^,^10^,^12^,^13^ FICBs are given by emergency physicians (EP) and residents without complications.^8^,^9^,^11^,^14^

Prior studies have shown good results from FICB in hip-fracture patients. These studies, however, all have small numbers, use different cut offs in evaluating pain, and pain scores are usually documented for short periods. Our objective was to investigate the success rate of administration of FICB in a busy Dutch ED with a standardized cut off for pain and a follow up of eight hours. The additional component in this busy ED is the always-rotating interns and residents. In the Netherlands, there have been no studies performed which investigated FICB in hip-fracture patients. Our study was initially designed in anticipation of a randomized double blind placebo-controlled trial (RCT).

## METHODS

### Study Design

This prospective pilot study was carried out on hip-fracture patients, presenting to the ED of the OLVG, between September 2012 and July 2013. The institutional review board approved the study.

### Study Setting and Population

Eligible patients were over the age of eighteen with clinical or radiological signs of a hip fracture. They presented with an intact cognitive status on admission and were able to give informed consent. We eliminated patients from the study if they displayed any cognitive impairment such as dementia or delirium or when surgery was planned within one hour. Patients were also excluded if they had any known allergy to local anesthetics, if an infection at the injection site was present, or if there was a history of femoral bypass surgery or an elevated International Normalized Ratio (above 4.5), or if they were admitted by the orthopedic department instead of general surgery. In the Netherlands, hip-fracture patients are treated by orthopedic and surgical teams. In the ED, patients are assigned to a specialty team based on day of the week rather than fracture type. During this study, for practical purposes, we only paired up with the surgical team. We included patients in the study when they met all inclusion criteria.

### Study Protocol

The study investigators (LG, MZ, JR) trained EPs and residents with different levels of experience in how to perform the FICB. The form of teaching was through lectures and assisting physicians in performing the procedure until competency was demonstrated. EPs and residents were supervised three times in the performance of FICB until the study investigators felt they were competent to perform it independently. The lecture was comprised of background information, anatomy, technique, drug interactions and study protocol. The study investigator gave multiple demonstrations in the ED. EPs and residents were encouraged to ask any questions. All physicians were provided with a pocket card containing information about FICB, such as procedure protocol and details of the medications to be administered.

A standardized FICB technique was used on all eligible patients. The patient was placed in a supine position, the inguinal ligament was identified and the femoral artery was palpated. After the skin was cleaned with chlorhexidine, FICB was given with a SonoPlex Stim cannula 22G×50mm needle without the use of a nerve stimulator. The needle was inserted perpendicular to the skin at a point 1cm below the juncture of the lateral and medial two-thirds of a line that joins the pubic tubercle to the anterior superior iliac spine. The needle was inserted until a loss of resistance was felt as the fascia lata was passed, and further advanced until a second loss of resistance occurred when the fascia iliaca was pierced (often described as “two pops”). This technique was first described by Dalens et al.^6^ We ruled out intravascular injection by aspiration. The dosage was 2mg levobupivacaine per kg, with a maximum of 175mg. When patients were under 75kg, we diluted the levobupivacaine 0.5% with saline solution to achieve an injection volume of at least 30 ml. We chose levobupivacaine as the local anesthetic due to its proven safety, widespread availability, and long-lasting effects.^15^ The FICB was never re-administered: it was a single shot.

All of the patients had a peripheral intravascular access and were supplied with oxygen as needed. Electrocardiogram, non-invasive blood pressure and oxygen saturation were monitored and documented. Any change in cognitive functioning after the administration of the FICB was recorded in each patient’s chart. EPs and residents were interviewed by the investigator after finishing two or more FICBs to ascertain whether they felt they had mastered the technique. Nurses were interviewed and asked to give feedback on whether the FICB was successful in patients’ pain management. Other collected data included patient demographics and type of fracture.

### Data Analysis

Nurses performed pain assessment during the first hour in the ED and until eight hours on the surgical ward. We used a 10-point numeric rating scale (NRS). This assessment was done before any medication was given, during FICB, and after the FICB was performed at 30, 60, 120, 240 and 480 minutes. The need for and use of supplemental analgesia was documented. Patients received a short-acting opioid in the ambulance or in the ED if pain was rated NRS≥7. In our analysis, we rated these patients “positive” for one hour because these short-acting opioids are usually active for more or less one hour. If patients received a long-acting opioid, for example morphine, we rated them “positive” for opioids for the duration of four hours. All patients received one gram of acetaminophen and 50 mg of diclofenac (Voltaren, an NSAID), when there were no contra indications for the use of these medicines.

For the analysis, we put patients in two groups: group one were patients who achieved a clinical reduction in pain without additional opioids. Group two were patients who achieved a clinically relevant reduction in pain with the supplemental use of opioids. A clinically relevant reduction of pain was reached when patient’s level of pain was lowered by ≥35% when the initial pain score was ≥6 (moderate pain) on the NRS. For patients with severe pain (NRS≥8) a decrease of ≥45% was regarded as clinically meaningful according to Soledad Cepeda and colleagues.^16^

We used a one-way repeated measures analysis of variance to assess the change in NRS in time. A p<0.05 was considered statistically significant. Statistical analyses were performed using SPSS 18.0 software package for Windows (SPSS Inc, Chicago).

## RESULTS

**Figure 1:**
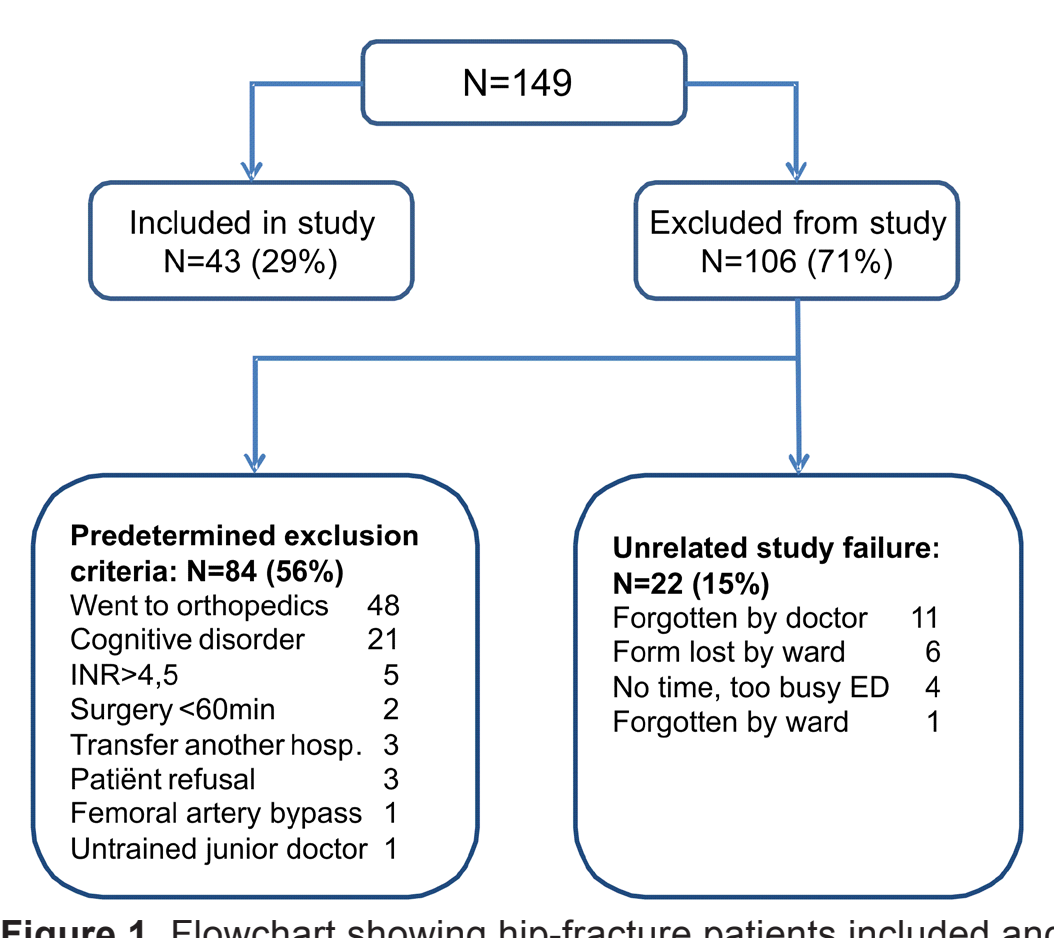
Flowchart showing hip-fracture patients included and excluded from study-analysis. *INR*, international normalized ratio; *ED*, emergency department

Figure 1 shows the study flowchart. In total, 149 hip-fracture patients were screened for inclusion in the study. Forty-three patients were included in the study, while 84 patients met predetermined exclusion criteria. Twenty-two patients dropped out due to unrelated study failure.

Forty-three patients were included in this study: 22 women, 21 men, with a mean age of 76 years. Nineteen patients had a femoral neck fracture and the other 24 patients had a pertrochanteric or subtrochanteric fracture. There were no patients with an ASA-score of 4 or 5. Thirty-three percent of patients showed an ASA-score of 3; 49% of patients showed an ASA score of 2. Only 12% of patients had a history of diabetes.

**Figure 2:**
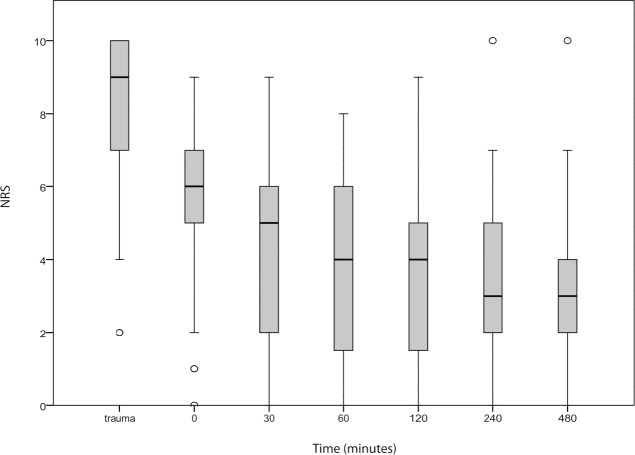
Boxplots showing overall reduction in pain (NRS) during admission (in time). *NRS,* numeric rating scale

Figure 2 shows the reduction in pain during admission. Overall, it is clear that patients experienced less pain after the FICB (p=0.04). This reduction in pain was studied in conjunction with the use and non-use of opioids.

**Figure 3:**
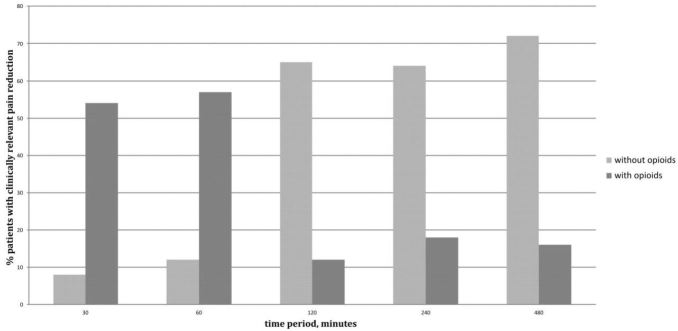
Percentage of patients with a clinically relevant reduction in pain. All values are reported in number (%). Time period is minutes after block placement. Missing values were respectively: 6, 8, 9, 15, 18, which means: numbers of documented pain scores at time 30 minutes were for 37 patients; after 60 minutes for 35 patients; after 120 minutes for 34 patients, after 240 minutes for 28 patients and 480 minutes after block placement pain scores are documented for 25 patients. Clinically relevant reduction in pain was reached when patient’s level of pain was lowered by ≥35% when the initial NRS was between 6–8 (moderate pain). For patients with severe pain (NRS≥8) a decrease of ≥45% was regarded as clinically meaningful. *NRS,* numeric rating scale

Differences in pain were registered from 30 minutes to 480 minutes after the FICB, which are represented in Figure 3. Analyzing all patients 120 minutes after the FICB, there was a clinically relevant reduction in pain in 76% of patients. At this moment, 12% showed this reduction with the use of opioids. However, 65% achieved this reduction without additional opioids. After 240 minutes, this clinically meaningful reduction was achieved in 82% (in total) where 64% did not receive any opioids (these patients ended up in group one). After 480 minutes, this goal was reached in 88% where 72% of patients achieved this reduction without additional opioids (again, this is called group one). In 16%, this reduction was achieved with additional opioids, such as morphine (group two).

The FICB technique was performed with minimal risk to patients. The levobupivacaine was given at a safe distance from the neurovascular bundle. During this study 17 different residents were responsible for 34 FICBs, while EPs were responsible for the remaining nine. Close observations of the patients’ vital signs and cognitive function increased the safety profile of the procedure by allowing early detection of systemic toxicity. No adverse events were reported. FICB was easy to perform and required minimal training. They found it easy to master the technique. EPs and residents performed the procedure successfully within less than five minutes of instruction.

Nurses from the department of general surgery were enthusiastic because they believed they could take better care of patients after FICB placement. After this positive feedback we interviewed four nurses randomly. They were all very satisfied after block placement since they found an improvement in care when the patient received a FICB. Although we did not measure pain levels during movements such as transfers, most patients indicated experiencing no pain during transfers.

## DISCUSSION

FICB seems to be a safe and practical method for reducing pain in patients with a hip fracture. FICB has been reported to provide effective pain relief in hip-fracture patients when performed by anesthesiologists, without the use of a nerve-stimulator or ultrasound, and without causing major side-effects.^7^,^12^,^13^

The aim of this study was to investigate the safety profile and success rate in a busy ED in the Netherlands with rotating physicians and time pressures, as an alternative or additive to conventional analgesia for hip-fracture patients. In this study, we achieved clinically relevant differences in pain in 76% of 34 documented patients at 120 minutes and in 88% of 25 documented patients at eight hours. Our results are comparable to those published in earlier studies, in which the block was performed by non-anesthesiologists with a success rate of 70–80%.^9^,^14^ Elkodair considered a difference of three points or more from the patient’s baseline to be clinically meaningful while we used 35% or even 45%, regarding to Soledad Cepeda and colleagues. According to Soledad Cepeda it is a variable that depends of the baseline NRS^16^ instead of a fixed number.

Anesthesiologists routinely place nerve blocks for pain control in the pre- and post-operative period, but have traditionally used nerve stimulators to guide their placement. Most EPs do not routinely use nerve stimulators but are increasingly trained in ultrasonography. There is good evidence to show that peripheral nerve blocks performed with ultrasound guidance can be placed with great success. However, with a success rate of 76–88% we achieved very good results and ensured that our EPs and residents performed the loss of resistance technique correctly. This increases practicality in our busy ED as it avoids the need for ultrasound scanners. Most Dutch EPs have no access to an ultrasound machine in their EDs and it takes less time to perform a FICB without ultrasound.

This study shows a clinically relevant reduction in NRS in 62% of patients after 30 minutes (Figure 3), which is due to the effect of the FICB or to short-acting opioids. Because these opioids wear off after one hour, we could at least tell something about FICB’s effect after two hours, when it is certain there is no effect from short-acting opioids. Two hours after FICB placement, there was a clinically meaningful decrease in pain in 65% of patients, which is very likely due to the FICB effect since these patients didn’t use any opioids in the given timeframe. In this teaching hospital in the Netherlands, hip-fracture patients stay for one to two hours in the ED, after which they are brought to the department of general surgery, the orthopedic ward or to the operating room. So for two-thirds of the remaining patients, FICB seems to be a useful intervention. Moreover, after eight hours this effect is even more pronounced because after this period, almost three-quarters of patients showed a clinically relevant reduction in pain without the use of opioids. Satisfaction scores among them will be higher since they will need less or even no opioids at all, which are known to make patients feel nauseous or cause them to vomit.

Our recommendation is to give hip-fracture patients a FICB in the ED, but because levobupivacaine is a long-acting anesthetic, we have to bear in mind that additional short-acting opioids are possibly needed in the ED. Levobupivacaine seems to be more helpful in patients who have to wait four to eight hours for surgery. For ED patients, the use of lidocaine could be more helpful since onset of effect is usually within half an hour. Further research is needed and one of our recommendations is to investigate the use of lidocaine in FICB because this is a short-acting anesthetic and its effect will be more pronounced in the ED.

This pilot study ended after nine months because of reasons indicated below. Furthermore, willingness from personnel to start with the intended randomized placebo controlled double-blind study was low, due to very promising results. FICB was implemented in the protocol of our department.

## LIMITATIONS

The dropout rate was significant and mainly due to pre-defined exclusion criteria. During one shift, there was no EP available who could perform this technique. In the beginning of the study, doctors forgot to include patients in the study protocol. Unfortunately study forms were lost in six cases, probably during the transfer to another ward. For example, when patients went to the cardiology ward for cardiac problems and were thereafter brought to the department of general surgery, the forms weren’t traceable. In four cases it was too busy in the ED, so patients went upstairs before they were included in the study. And in one case, nurses from the department of general surgery forgot to fill in the form.

Unfortunately not all measurements of pain scores were performed. A possible explanation could be that nurses of the department of general surgery forgot to fill in the pain scores because patients didn’t complain about pain after administration of the FICB, or because the nurses may have been too busy to fill in the forms. The use of an independent research nurse would be a good option to minimize patient drop out.

We did control the amount of conventional pain treatment used before and after the FICB, but incidentally the patient was given morphine instead of fentanyl, which may have influenced the level of pain. To compensate this problem we measured pain at different time intervals, even after eight hours after block placement. The decrease in pain at four (and eight) hours after the FICB was likely due to the analgesia provided by the FICB, as this was beyond the scope of the effect of morphine.

## CONCLUSION

In a busy ED with rotating residents of varying levels of experience, fascia iliaca compartment block seems to be an efficient, safe and practical method for reducing pain in patients with a hip fracture. In two hours there was a clinically meaningful decrease in NRS in 76% where 65% of patients achieved this reduction without the use of opioids. Even after eight hours this reduction was achieved in 88% of patients from whom 72% didn’t needed opioids. No adverse events were reported. Because of small numbers and the lack of a control group, the investigator aims to collect more data to answer questions on safety and efficiency.

## References

[1] Orosz GM, Magaziner J, Hannan EL (2004). Association of timing of surgery for hip fracture and patient outcomes. JAMA.

[2] Chau DL, Walker V, Pai L (2008). Opiates and elderly: use and side effects. Clin Interv Aging.

[3] Morrison RS, Magaziner J, Gilbert M (2003). Relationship between pain and opioid analgesics on the development of delirium following hip fracture. J Gerontol A Biol Sci Med Sci.

[4] Candal-Couto JJ, McVie JL, Haslam N (2005). Pre-operative analgesia for patients with femoral neck fractures using a modified fascia iliaca block technique. Injury.

[5] Capdevila X, Biboulet P, Bouregba M (1998). Comparison of the three-in-one and fascia iliaca compartment blocks in adults: clinical and radiographic analysis. Anesth Analg.

[6] Dalens B, Vanneuville G, Tanguy A (1989). Comparison of the fascia iliaca compartment block with the 3-in-1 block in children. Anesth Analg.

[7] Foss NB, Kristensen BB, Bundgaard M (2007). Fascia iliaca compartment blockade for acute pain control in hip fracture patients: a randomized, placebo-controlled trial. Anesthesiology.

[8] Godoy MD, Iserson KV, Vazquez JA (2007). Single fascia iliaca compartment block for post-hip fracture pain relief. J Emerg Med.

[9] Hogh A, Dremstrup L, Jensen SS (2008). Fascia iliaca compartment block performed by junior registrars as a supplement to pre-operative analgesia for patients with hip fracture. Strategies Trauma Limb Reconstr.

[10] Kim HS, Kim CS, Kim SD (2011). Fascia iliaca compartment block reduces emergence agitation by providing effective analgesic properties in children. J Clin Anesth.

[11] Wathen JE, Gao D, Merritt G (2007). A randomized controlled trial comparing a fascia iliaca compartment nerve block to a traditional systemic analgesic for femur fractures in a pediatric emergency department. Ann Emerg Med.

[12] Yun MJ, Kim YH, Han MK (2009). Analgesia before a spinal block for femoral neck fracture: fascia iliaca compartment block. Acta Anaesthesiol Scand.

[13] Lopez S, Gros T, Bernard N (2003). Fascia iliaca compartment block for femoral bone fractures in prehospital care. Reg Anesth Pain Med.

[14] Elkhodair S, Mortazavi J, Chester A (2011). Single fascia iliaca compartment block for pain relief in patients with fractured neck of femur in the emergency department: a pilot study. Eur J Emerg Med.

[15] Burlacu CL, Buggy DJ (2008). Update on local anesthetics: focus on levobupivacaine. Ther Clin Risk Manag.

[16] Cepeda MS, Africano JM, Polo R (2003). What decline in pain intensity is meaningful to patients with acute pain?. Pain.

